# Stereological Method for Assessing the Effect of Vitamin C Administration on the Reduction of Acrylamide-induced Neurotoxicity

**DOI:** 10.29252/nirp.bcn.9.1.27

**Published:** 2018

**Authors:** Hengameh Dortaj, Maryam Yadegari, Mohammad Hosseini Sharif Abad, Abolghasem Abbasi Sarcheshmeh, Morteza Anvari

**Affiliations:** 1. Department of Anatomy and Cell Biology, Faculty of Medicine, Shahid Sadoughi University of Medical Sciences and Health Services, Yazd, Iran.

**Keywords:** Acrylamide, Cerebellum, Vitamin C, Stereology, Neurotoxicity

## Abstract

**Introduction:**

Acrylamide (ACR) consumption is increasing all over the world. There are some evidence on the literature about its neurotoxic effect on mature animals, but the effects of ACR on postnatal development have been less studied. The purpose of this study was to evaluate the effects of ACR on development of cortical layer, white matter, and number of Purkinje cells of the cerebellum in rat newborns.

**Methods:**

This study was carried out on 20 female Wistar rats (average weight: 180 g, aged: two months). The rats were divided into four groups. Pregnant rats were orally fed with ACR 10 mg/kg and vitamin C 200 mg/kg. In this study, 6 infants of each group (weighting 32–35 g) were randomly selected at day 21 after birth and placed under deep anesthesia and transcardial perfusion. Their cerebellums were fixed and histopathological changes were evaluated with Hematoxylin and Eosin (H&E) staining and cresyl violet method. The volume of cerebellar cortical layers and number of Purkinje cells were investigated by Cavalieri’s principle and physical dissector methods. The obtained data were analyzed by 1-way ANOVA and LSD test using SPSS. P<0.05 considered as statistically significant.

**Results:**

The results showed that newborns of ACR-treated female rats have decreased cerebellar weight (P≤0.05) and lower than average number of Purkinje cells (P≤0.001). ACR also decreased the volume of granular and molecular layer and increased the volume of white matter. While the results showed decreased in white matter volume in vitamin C group (P≤0.001).

**Conclusion:**

ACR induces structural changes in the development of the cerebellar cortical layers in rat newborns, but these changes may be prevented by vitamin C as an antioxidant.

## 1. Introduction

The cerebellum is an ideal system to study pattern formation in the Central Nervous System (CNS) because of its simple cytoarchitecture and regular organization of neural circuitry and folds (Trenkner, 1977). Because of the active development, neonatal life is a very sensitive period that could be easily affected by endogenous or exogenous factors. Acrylamide (ACR), CH_2_=CH-CONH_2_, is a small water-soluble vinyl monomer and hydrophilic molecule with so many chemical and industrial applications (Friedman, 2003). ACR polymer is used extensively in laboratories for gel chromatography and also in modern chemical technology, like filtration in water treatment or processing industries like paper mills (Singhal et al., 2008).

ACR is present in foods such as fried potato, cookies, and crackers that are prepared at very high temperature as a result of Maillard reaction between sugar and amino acids (e.g. asparagine) (Ayvaz, 2014). ACR has been classified as a probable carcinogen and is a well-documented neurotoxic in both humans and animals (Tilson & Cabe, 1978). Subchronic, low-level exposure of humans to ACR produces neurotoxicity characterized by ataxia and skeletal muscle weakness of the feet (Shuming, Jilin & Xichun, 2009).

Previous morphological studies suggest that both humans and experimental neurotoxicity were mediated by cerebellar Purkinje cell injury through degeneration of distal axon in the peripheral and central nervous system (Lehning, Balaban, Ross & LoPachin, 2003). Purkinje cells have an inimitable persistence and exhibit unparalleled regenerative capabilities within the central nervous system. Their response to cell injury is unique among most neurons and can show degenerative, compensatory, and regenerative mechanisms (Lehning et al., 2003).

Axonal remodeling of Purkinje cells clearly increases in the disease. A disease-related increase in the frequency of Purkinje cell fusion and heterokaryon formation in ataxia cases have been reported (Lehning et al., 2003).

Vitamin C is important as an antioxidant in cellular function (Volterra, Trotti, Tromba, Floridi, & Racagni, 1994). This antioxidant plays a pivotal role in neutralizing free radicals induced by oxidative recompense to lipids and lipoproteins in various cellular compartments and tissues (Volterra et al., 1994). CNS neurons contain some of the highest vitamin C concentration in tissues (Martin, Janigian, Shukitt-Hale, Prior, & Joseph, 1999). Vitamin C is capable of crossing the blood-brain barrier and removing free radicals from both inside and outside the cells (Agus et al., 1997). Quantitative morphology of CNS has been recently undergone major development (Jacobson, 2013). Stereology methods have been successfully applied to neuromorphological research (Schmitz & Hof, 2005). The data on the effect of ACR on the postnatal development of cerebellum is rare (Kopańska, Lukáč, Kapusta, & Formicki, 2015). So the aim of this study was to investigate the histopathological and stereological assessment for estimating the effect of vitamin C administration on the reduction of ACR induced neurotoxicity.

## 2. Methods

Chemical: ACR (99% pure) and formaldehyde solution was purchased from Sigma chemical company. Vitamin C was purchased from Hakim Pharmaceutical Company, Iran.

### 2.1. Experimental procedures

The Ethics Committee of Shahid Sadoughi University of Medical Sciences approved all study procedures. In the present study, 20 young female Wistar rats were used and marked. They were housed 5 per cage and fed standard rodent pellet diet. Drinking water and rodent laboratory food were available ad libitum. The animal room was kept at 20ºC–25ºC and 50% humidity under 12:12 h light-dark cycle.

Female rats were mated with males (2:1) in each cage. In the next morning, a positive sign of mating was confirmed by sperm-positive vaginal smears and the presence of copulatory plugs. The presence of sperm in the vaginal smear was determined at Day zero (D0) of gestation. The pregnant mothers were randomly labeled into 4 groups. Group A consisted of pregnant rats which were given distilled water (control group). Group B included pregnant rats which were given ACR from day 7 of gestation (D7). Group C were pregnant rats which received vitamin C from D7 of gestation. Finally, group D comprised pregnant rats which were given ACR+vitamin C from D7 of gestation. ACR was dissolved in distilled water and administered orally to pregnant rats at a dose of 10 mg/kg/day and vitamin C 200mg/kg/day (Raju et al., 2013).

### 2.2. Animal perfusion

To avoid physical injury, rats’ cerebellums were fixed by perfusion before exposing them out of the skulls. Newborns were undergone transcardial perfusion 21 days after birth. The perfusion solution was 4% formal-dehyde in 0.1 M phosphate buffered saline. First, the animals were deeply anesthetized with diethyl ether. Then they were placed in supine position. The thoracic cavity was opened by parasagittal skin incision. To perform perfusion, right atrium was opened to permit the blood and the fixative leave the body. Next, the rats were per-fused with perfusion solution (14% of total body weight) for 10–15 minutes until the fluid that comes out of the rat became clear. After perfusion, the skull was cut and the entire cerebellum was weighed (Villringer, Them, Lindauer, Einhaupl, & Dirnagl, 1994).

### 2.3. Histology assessment

For histological analysis of newborn cerebellum, the whole cerebellum was dissected and inserted into Bovine fixative at room temperature overnight for 2 days and then transferred to 70% ethanol. For light microscopic study, after fixation the samples were dehydrated by alcohol, cleared in xylene, and then embedded in paraffin wax. Sections (5 μm thick) were stained with Hematoxylin and Eosin (H&E) and with cresyl violet method to identify neurons. Stereological analysis and the grids were used to determine the volume of the cerebellum layers and number of Purkinje cells (Tunc et al., 2006).

### 2.4. Stereology assessment

#### 2.4.1. Cortex volume

The volume of the cerebellum was estimated by using the Cavalieri’s principle method (Heidari & Mahmoudzadeh-Sagheb, 2012). Cavalieri’s method allows for the estimation of total volume from area on a systemic-random sampling of sections through the objects. The grid points were randomly superimposed over each section under a dissecting microscope and the points falling on the cerebellum were counted. On the average, 180–200 hits per cerebellum was counted. Volume of the cerebellum was determined by applying the following formula:

V=∑P×t×d×a/p,

where t is the fixed thickness of the slabs of the cerebellum (5 μm), a/p is the area associated with each point of the grid, and ∑P refers to the total number of points that hit with the cerebellum cortex (Heidari & Mahmoudzadeh-Sagheb, 2012). An average of 10 sections was counted per cerebellum. The magnification of cerebellum using a light microscope was 100x for embryonic cerebellums and 40x for newborns. At this magnification, the differentiation between cortex and white matter can be performed.

### 2.5. Number of Purkinje cell

#### 2.5.1. Numerical density of Purkinje neurons

The numerical density of neurons was counted by the physical dissector method. The dissector consists of a pair of serial sections, a “look-up” section and a “reference” section, with fix distance “h.” According to the dissector method, neurons to be counted are those that can determine in the reference section and not on the lookup section (West, 1999). In the present study, a randomly selected area of cerebellar cortex from the reference section was taken for staining process. An equivalent region of cerebellar cortex from the look-up section was also photographed. On the photographs, the transparent point-counting grid was randomly thrown on the reference section and after that it was superimposed on the same region of look-up section. Then, neurons within the grid and those intercepted by the right vertical and top grid bars (ac-ceptance line) were counted but those intercepted by the left vertical and bottom bar (forbidden line) were not included in the count. Then, the numerical density in any region was calculated using the formula (Behnam-Rasouli, Nikravesh, Mahdavi-Shahri & Tehranipour, 2000).

N=∑Q/af.h∑P,

where N refers to numerical density, Q is the number of cells seen on the reference section not on the look-up section, a/f is the area associated with each frame, h denotes the distance between sections, and P is the number of frames associated points hitting the tissue.

#### 2.5.2. Total number of Purkinje cells

After estimation of the number of neurons per unit volume (numerical density) and volume of cerebellar cortex, the total number of Purkinje cells in cerebellum is estimated. The total number of neurons was calculated by the following mathematical equation (West, Slomianka & Gundersen, 1991).

Nn=N.V,

where Nn is the total number of neurons, N refers to numerical density of Purkinje neurons, and V is the volume of cerebellar cortex.

### 2.6. Statistical analysis

Statistical significance was determined by 1-way Analysis of Variance (ANOVA) to compare the normal vs. experimental groups. Post hoc test for this estimation was Least-Square Distance (LSD). Statistical analysis was performed using SPSS (version 19). A probability of less than 0.05 was considered a significant difference between group means.

## 3. Results

**Figure 1.**
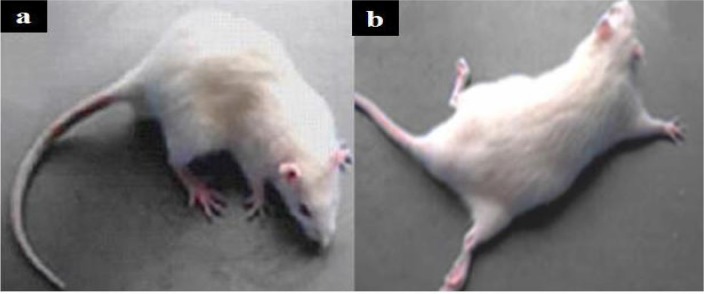
Signs of ACR anatomical changes a: There were not any changes in hind-limbs of the control group; b: Weakness of hind-limb muscles and paralysis were observed in ACR-treated newborns after birth.

Signs of ACR toxicity; weakness of hind-limb muscles and paralysis were observed in the newborns from ACR-treated rats (Figure 1). Comparative histological changes in 4 groups and light photomicrographs in H&E staining of Purkinje cells in neonatal period by using grid and physical dissector are shown in Figure 7. Comparative histological and stereological changes in 4 groups using cresyl violet staining showed for estimating number of Purkinje cells (Figure 8) and volume of cerebellum (Figure 9).

### 3.1. Volume of molecular layer in newborn

**Figure 2.**
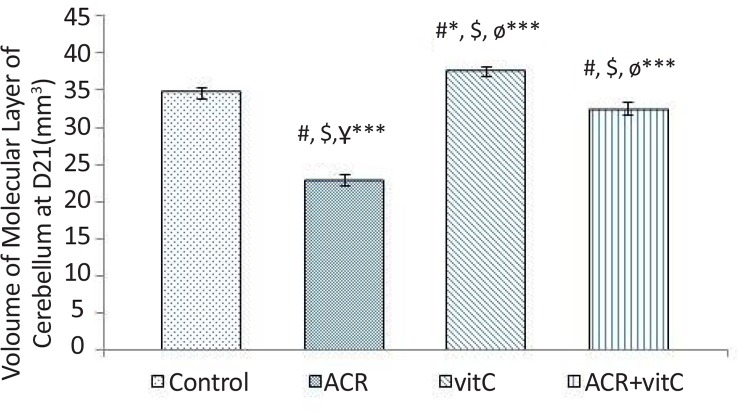
Volume of molecular layer in newborn rats (mm^3^) #: Estimating the comparison between the control and other groups; $: Comparison between vitamin C group and the other groups; Ұ: Comparison between ACR+vitamin C group and other groups; Ф: Comparison between ACR and other groups; * (P<0.05); *** P<0.001

Stereological investigation in the volume of molecular layer 21 days after birth, in ACR (P<0.001) and ACR+vitamin C groups (P=0.011) was significantly decreased compared with the control group, but was significantly increased (P<0.001) in the vitamin C group vs. control group (Figures 2, 9).

### 3.2. Volume of granular layer in newborn

**Figure 3.**
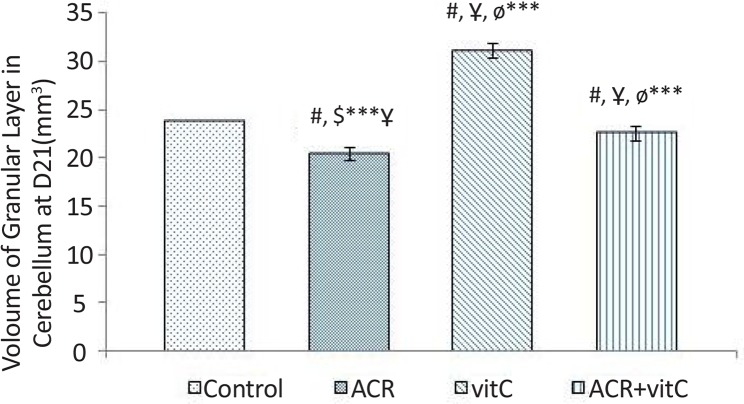
Volume of granular layer at D21 of newborn rats (mm^3^) #: Estimating the comparison between the control and other groups; $: Comparison between vitamin C group and other groups; Ұ: Comparison between ACR+vitamin C group with other groups; Ф: Comparison between ACR group with other groups; * P<0.05; *** P<0.001

Stereological investigation showed that the volume of granular layer at day 21 after birth, in ACR and ACR+vitamin C groups was significantly decreased compared with the control group (P<0.001), but this reduction in volume of granular layer in ACR group compared to ACR+vitamin C group was significant (P=0.005). Volume of the granular layer in vitamin C group vs. control group was significantly increased (P<0.001) (Figures 3 and 9).

### 3.3. Volume of white matter in newborn

**Figure 4.**
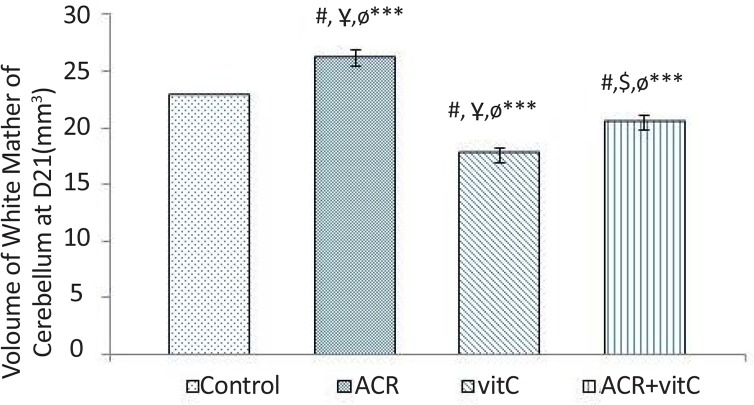
Volume of cerebellum white matter at D21 in mm^3^ #: Estimating the comparison between the control group and other groups; $: Comparison between vitamin C group and other groups; Ұ: Comparison between ACR+vitamin C group and other groups; Ф: Comparison between ACR group with other groups. *** P<0.001

Stereological investigation showed that volume of white matter 21 days after birth, in ACR and ACR+ vtamin C groups was significantly increased compared with the control group (P<0.001). White matter volume in vitamin C group vs. control group was significantly decreased (P<0.001) (Figures 4 and 9).

### 3.4. Whole volume of cerebellum

**Figure 5.**
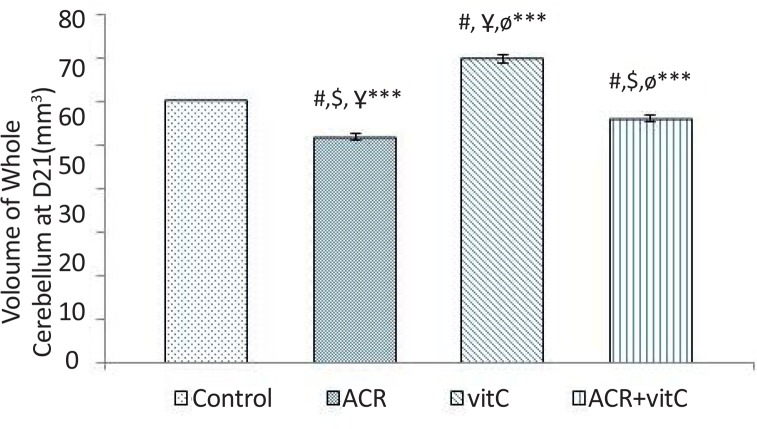
Volume of whole cerebellum at D21 (mm^3^) #: Comparison between the control group and other groups; $: Comparison between vitamin C group and other groups; Ұ: Comparison between ACR+vitamin C group and other groups; Ф: Comparison between ACR group and other groups; *** P<0.001

Stereological investigation showed that the total volume of the cerebellum at day 21, in ACR and ACR+vitamin C groups was significantly decreased compared with the control group (P<0.001). While the total volume of the cerebellum in vitamin C group vs. control group was significantly increased (P<0.001) (Figures 5, 9).

### 3.5. Number of Purkinje cell

**Figure 6.**
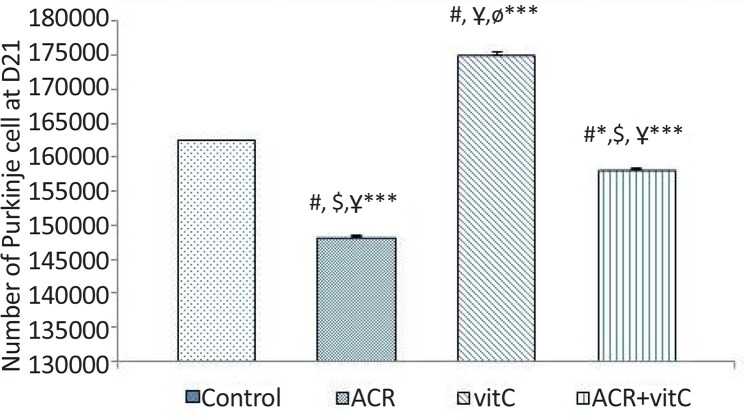
Number of Purkinje cell at D21 #: Comparison between the control group and other groups; $: Comparison between vitamin C group and other groups: Ұ: Comparison between ACR+vitamin C group and other groups: Ф: Comparison between ACR with other groups; * P<0.05; *** P<0.001

**Figure 7.**
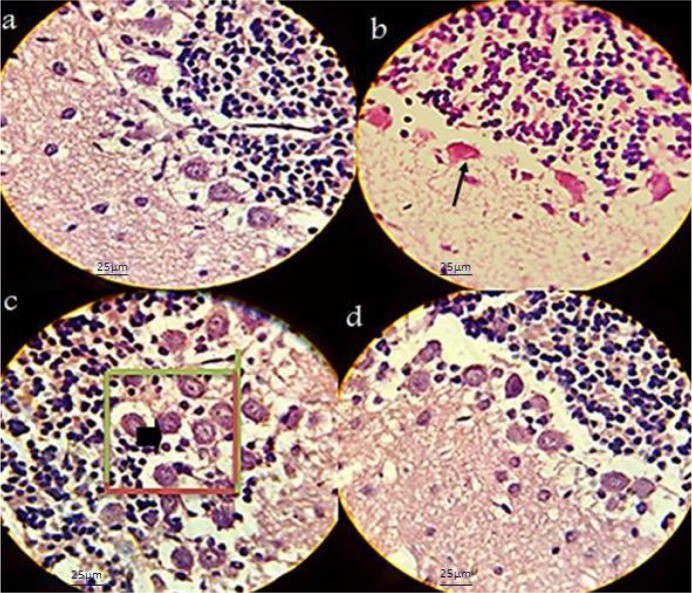
Light photomicrographs estimating the number of Purkinje cells in neonatal period by using grid and physical dissector H&Ex1000 Forbidden and allowed sides to count red and green are separated. Decreasing in Purkinje cells in the ACR group (arrow) and increasing cells seen in the vitamin C group (arrow head). a: Control; b: ACR; c: Vitamin C; d: ACR+vitamin C; Scale bar: 25 μm

**Figure 8.**
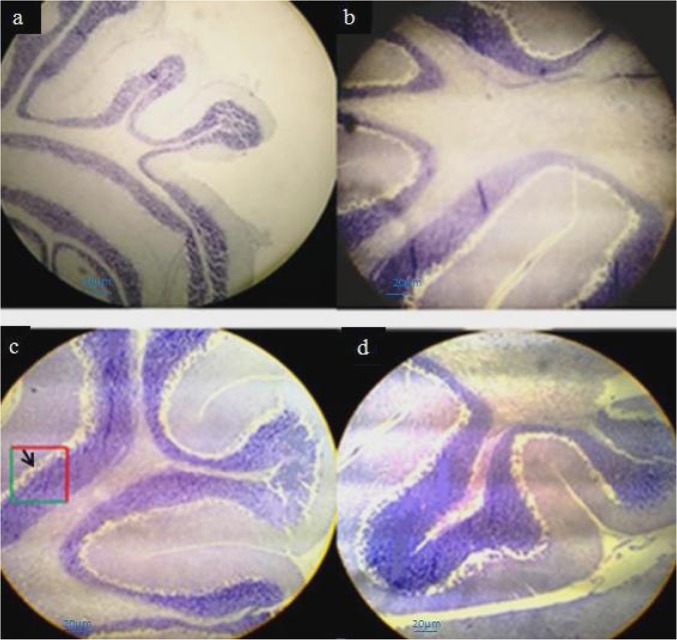
Light photomicrographs estimating the number of Purkinje cells in neonatal period by using grid and physical dissector cresyl violet staining (×100) Forbidden and allowed sides to count red and green are separated. Decreasing in Purkinje cells in the group of ACR and the number of Purkinje cell was increased in the vitamin C group (arrow). a: Control; b: ACR; c: Vitamin C; d: ACR+vitamin C; Scale bar: 20 μm

The results showed that ACR affected newborns and decreased the average number of Purkinje cells (P≤0.001) and vitamin C increased the average number of Purkinje cells (P≤0.001). In ACR+vitamin C group, this reduction was significant compared to the control group (P=0.006) (Figures 6–8).

**Figure 9.**
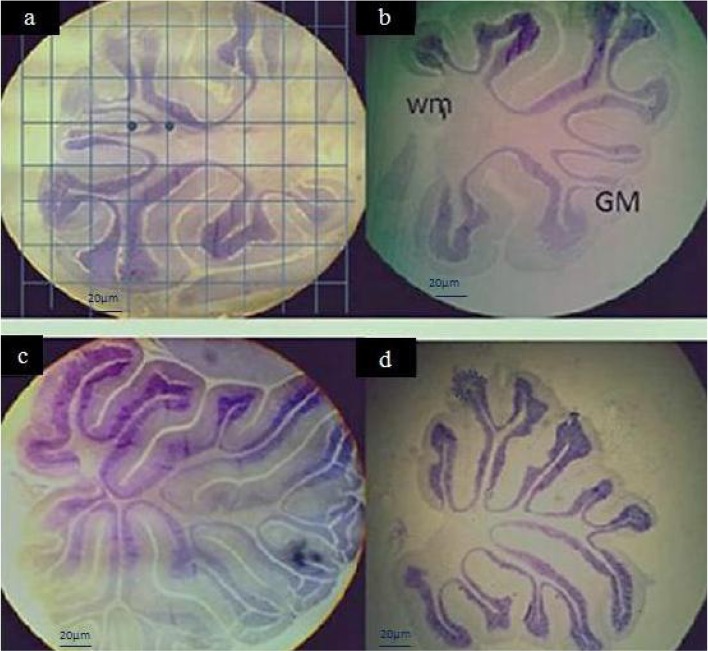
Light photomicrographs estimating volume of Gray Matter (GM), White Matter (WM), and whole cerebellum in neonatal period by using grid and physical dissector cresyl violet staining (×4) Cerebellar white matter volume increases in ACR group and gray matter increase in vitamin C group comparing with the control group. a: Control; b: ACR; c: Vitamin C; d: ACR+vitamin C; Scale bar: 20 μm

## 4. Discussion

In the present study, administration of ACR caused a significant decrease in molecular, volume of granular, whole cerebellum volume, and average number of Purkinje cells. It also increased volume of white matter cerebellum in newborn measured 21 days after birth. In this study, the stereological technique, H&E and cresyl violet were used to assess changes in the cerebellum. The information on the effects of tissue damage caused by eating fried starch-containing acrylamide during pregnancy on the development of fetus nervous system is scarce. Thus this study aimed to fill this information gap.

The previous studies demonstrated that ACR acts as a neurotoxic in both laboratory animals and humans (LoPachin, 2005). In the current study, the volume of cerebellar cortex was found to be shrunk in both ACR and ACR+vitamin C treated animals. Thus, it appears that exposure of animals to ACR might affect the cerebellum development. Impaired growth and development in embryonic period may be due to the toxic effects of ACR crossing through placenta during pregnancy (Duarte-Salles et al., 2012). Stereological analysis showed a decrease in the volume of molecular and granular layer of the cerebellum and in-crease in the volume of white matter in newborns in ACR and ACR+vitamin C groups.

Reduction in molecular and granular layer volume in these two groups may be the result of the process that damages nerves terminals in this region. Increase in the volume of white matter in ACR and ACR+vitamin C groups may be due to increase in myelin synthesis to compensate the reduction of gray matter (Gold, Griffin, & Price, 1985). Development of nerve cells in laboratory animals and humans occur over a period of time before birth and continues in the cerebellum, even after birth. According to most studies, nerve cells are made in the mid-term pregnancy and then began to migrate to their final locations (Clendeninn, Petraitis & Simon, 1976; Sidman & Rakic, 1973). The results showed that administration of ACR to pregnant rats would result in a significant reduction in the average size of the cerebellum in new-borns compared to the control group. This result was in agreement with previous findings that showed the sensitivity of the brain cells in neonatal nutrition during pregnancy and lactation by ACR (Zeisel, 2004; El-Sayyad et al., 2011).

Research studies have shown that 10%to 15% of ACR in the diet of pregnant women are transmitted to the fetus through the placenta and more than 8.18 mg/L through the breast milk (El-Sayyad et al., 2011; Sörgel et al., 2002). Thus the concerns over the exposure of mothers to ACR in the food has been increased (Brantsæter et al., 2008). Evidence suggests that the binding proteins in the Central Nervous System (CNS) to produce the enzymes that compound with ACR play an important role in the neurotoxicity of ACR (Sakr, Badawy, El-Sayyad & Afify, 2011). Barber and colleagues reported that ACR during prenatal period induces biochemical disorders, oxidative stress, and changes in the structure of the cerebellum (Barber & LoPachin, 2004). This probably occurs due to increased Reactive Oxygen Species (ROS) levels in the tissues (Rydberg et al., 2003). Their result was in agreement with the results of the current study that ACR induces some changes in the structure of the cerebellum.

Antioxidants such as vitamin C act by binding with free radicals in tissues (Machlin & Bendich, 1987; Fang, Yang & Wu, 2002; Ou et al., 2010). The use of vitamin C in this study reduced the damaging effects of ACR. The key role of the cerebellum in balance and its formation during fetal life highlight the importance of research on the protection of children from embryonic period exposure to ACR. The idea that animal research, particularly those related to pharmaceuticals and environmental agents, may be a poor predictor of human experience is not new. Some limitations of animal model studies in human research lies in planning, conducting, and critically evaluating studies utilizing animal models. Despite their limitations, animal models remain fundamental in improving our understanding of human toxicology. In conclusion, the present study showed that the neurological damage of the cerebellum under the effect of ACR can be minimized by vitamin C; this may be due to its antioxidant and free radical scavenging activities.
